# Parental Engagement in Early Literacy: A Qualitative Exploration of Practices and Beliefs in Northern Mexico

**DOI:** 10.11621/pir.2024.0401

**Published:** 2024-12-01

**Authors:** Norma Beltrán-Sierra, Blanca Fraijo-Sing, Cesar Tapia-Fonllem

**Affiliations:** a University of Sonora, Hermosillo, Mexico

**Keywords:** early literacy, home literacy environments, preschool children, maternal beliefs, reading practices, writing practices

## Abstract

**Background:**

Developmental studies have shown that the home environment has a significantly influences subsequent academic performance by supporting the development of skills essential for the acquisition of competencies necessary for school life, including literacy skills. Children with limited early literacy proficiency often experience challenges in acquiring literacy skills. Family literacy promotes the development of skills necessary for their acquisition.

**Objective:**

The aim of this study is to identify and categorize literacy practices conducted at home, examine the participation of parents and children in reading and writing activities, and explore mothers’ beliefs on this subject.

**Design:**

For the present study, interviews were conducted with 102 mothers of preschool children in a city in northwestern Mexico. The approach for this study is qualitative and constructivist.

**Results:**

The study identified key dimensions: limiting environment, literacy interface, physical environment, interaction frequencies, and beliefs. These dimensions consistently highlight *shared time* as the main activity to promote reading and writing. However, the strategies deemed most relevant to language stimulation are primarily academic and place special emphasis on remediation and explicit teaching.

**Conclusion:**

Despite expressing interest in developing activities to encourage reading and writing at an early age, these activities often fail to align with skills and developmental period of preschool children. The adult-centered and formal view of literacy practices in the home may be enhanced by educating the parents about their children’s developmental stages and literacy needs.

## Introduction

*Early literacy* is defined as the nurturing of reading, writing, and language skills in children, beginning from birth and continuing before they start school (Debaryshe et al., 1996; Teale y Sulzby, 1986). In research, early literacy posits that everyday experiences during this developmental period provide the foundation for later language skill acquisition, reading and writing proficiency, as well as academic success and social integration (Adamson et al., 2021; Hassunah-Arafat et al., 2021; Loye et al., 2022; Teale & Sulzby, 1986). The role of literacy extends beyond reading and writing. From a social and cultural perspective, literacy enables individuals to actively participate in their communities. This involves more than just decoding sounds and symbols and encompasses reasoning, critical attitudes, and personal development (UNESCO, 2016). According to Vygotsky’s theory on socialization through language and interaction, and Bronfenbrenner’s ecological systems model, research on early childhood development highlights the importance significance of children’s environments and their interactions with more experienced individuals. Thus, the home environment and the role of mothers are particularly relevant during this stage of development.

### Early Literacy

To clarify the construct of initial literacy, McLachlan and Arrow (2017) offer critical perspectives. They advocate for the term *early literacy* over *pre-literacy* to emphasize that foundational knowledge necessary for learning to read typically emerges in early childhood and supports the acquisition of more conventional literacy skills. Previously, literacy was often understood as a set of reading, writing, and math skills; however, it is now recognized as encompassing *identification*, *comprehension*, *interpretation*, *creation*, and *communication* (UNESCO, 2023).

Children are continually involved in cognitive activities related to language use, whether oral or written, and often display reading and writing behaviors in informal settings. The development of listening, speaking, reading, and writing skills occurs concurrently and is closely interconnected (Teale & Sulzby, 1986).

### Parental Literacy Beliefs

Parental beliefs reflect socially constructed ideas about literacy and how individuals develop literacy, often shaped by parents’ own experiences (Debaryshe et al., 1996; Tsirmpa et al., 2021). These primarily influence child development indirectly through the actions caregivers take based on their beliefs in specific areas of parenting (Aram & Yashar, 2023; Hassunah-Arafat et al., 2021; Tsirmpa et al., 2021). Recent research highlights mothers’ and fathers’ beliefs as key factors shaping caregiver behaviors and potentially influencing the effectiveness of both adult- and infant-focused interventions (Batista Rocha & da Mota, 2023; Justice et al., 2020; Tanji & Inoue, 2023). Similarly, it is recognized that mothers’ and fathers’ beliefs affect their actions and behaviors with respect to the expectations held about their daughters and sons in several domains, including early literacy (Aram et al., 2016; Sajawandi et al., 2021). Therefore, mothers’ and fathers’ beliefs about reading and writing are correlated with children’s early literacy skills (Husain et al., 2011).

According to Parecki and Gear (2013) caregivers tend to reflect on their own literacy beliefs and behaviors, which influence the support they provide to infant development, especially if they have higher levels of education, recall positive experiences in their own literacy processes, or have participated in programs aimed at supporting the literacy process. Similarly, it has been recorded that mothers with a low level of education intervene less frequently in literacy promotion activities with their children. Consequently, they engage in interactive reading less frequently, running the risk of limiting their children’s exposure to language skills that foster reading and writing from an early age (Carneiro, et al., 2019; Kotrla Topic et al., 2020).

Likewise, caregivers’ beliefs often vary regarding their role in literacy instruction. Tsirmpa et al. (2021) note that some mothers and fathers believe it is their responsibility to actively support and promote their children’s literacy development, while others assert that this commitment belongs primarily to the school. According to these beliefs, mothers and fathers exhibit a variety of literacy practices, however, these practices may or may not lead them to believe that this responsibility falls within their role as caregivers (Anyikwa & Obidike, 2012; Aram et al., 2016; Bojczyk et al., 2016; Hume et al., 2015; Parecki & Gear, 2013; Sajawandi et al., 2021; Tsirmpa et al., 2021). Therefore, various scholars propose viewing literacy from a developmental perspective and explore the different contexts where they carry out knowledge exchange, with the family being the first and most important environment.

### Literacy Environments

McLachlan and Arrow (2017) point out that the study of early literacy developed in homes and early childhood settings is primarily aimed at documenting the process of acquiring and developing these practices from the earliest stages. This is done, in part, in order to understand why some children enter school with greater language skills related to reading and writing. It also highlights the relevance of examining the interaction between their family environments, early childhood contexts, and their access to various resources that promote literacy. One way to approach the study of this phenomenon and evaluate experiences is through the Home Literacy Environments (HLE) model (Lau & Richards, 2021; Rohde, 2015). HLE is defined by Kumalasari & Sugito (2020) as home-based experiences that help children learn a variety of subjects, where every object supports learning and includes people who are involved in contributing to knowledge. It encompasses a wide range of interrelated attitudes, activities, spaces, and resources, with different components of HLE linked to various aspects of literacy and language skills in preschool children (Lau and Richards, 2021; Rohde, 2015; Yeo et al., 2014).

Burgess et al. (2002) argue that the HLE is shaped by both the range of resources and opportunities accessible to children as well as the parental skills, abilities, attitudes, and resources that influence the availability of these opportunities. The author further introduces two concepts—Limiting Environment and Literacy Interface—to deepen the understanding of the HLE. The *Limiting Environment* suggests that a parent’s ability and willingness to provide literacy opportunities for their children are determined by the resources at their disposal. These resources encompass broader factors like social class, as well as specific parental characteristics such as intelligence, language and reading skills, and attitudes toward education. On the other hand, the *Literacy Interface* highlights the ways in which parents participate in activities that directly or indirectly expose their children to literacy experiences, such as shared reading, or convey the value they place on literacy, for instance, through their own reading habits.

Stimulating HLEs have been found to enhance opportunities for literacy development (Attig and Weinert, 2020; Ebert et al., 2020; Ergül et al., 2017; Karpava, 2021; Luo et al., 2021; Wang et al., 2021), and the creation of these spaces depends on a variety of environmental, psychological, and cultural factors, such as parental education level, socioeconomic status, time and resources available for practice, and household language background, to name a few (Attig and Weinert, 2020; Inoue et al., 2020; Kumalasari & Sugito, 2020; Niklas et al., 2020; Tanji and Inoue, 2023). These factors can promote or hinder the development of a rich linguistic repertoire and the use of language in various contexts that impact literacy (Karpava, 2021). Family literacy practices foster the acquisition of skills essential for academic life, while at the same time present improvements in cognitive level, school performance, oral language, reading process and socioemotional development of children (Kong & Yong, 2023; Pelosi et al., 2019; Wirth et al., 2022).

### Research objectives

In Mexico, a decrease in the reading population has been observed, especially in its printed format, according to the results of the National Institute of Statistics and Geography (Instituto Nacional de Estadística y Geografía, INEGI) in the reports *Module on Reading* published in 2022, 2023 and 2024. These reports indicate that, the lower the educational level, the lower the reading time; in general, men read a little more than women and on average only 30 percent consider understanding the text read, data that corroborate the lack of reading comprehension and the low scores in evaluations carried out by national and international agencies (OCDE, SEP, UNESCO). Additionally, it has been recorded that almost 70 percent of the reading population are encouraged to read both at home and at school, 16 percent only at school, 4 percent only at home and 9 percent say they have not received any type of motivational stimulus. The respondent population stated that the reasons for not reading most frequently are lack of time and lack of motivation or interest in reading according to INEGI (2022, 2023, 2024). It is only in the most recent survey conducted (2024) that a new series of questions are included, where they address literacy encouragement at home. including access to reading material, modeling by tutors, shared reading and encouraging library attendance (INEGI, 2024).

Considering the situation in Mexico, the purpose of this study is to analyze family beliefs about the literacy process at home based on the attitudes indicated in the questions regarding the creation of HLEs, such as the role of caregivers in the development of reading and writing skills, how language is stimulated, how literacy practices are approached, and the availability of materials and spaces for these activities. With these perceptions of literacy, it is possible to establish plans focused on undermining the deficiencies in the family environment and strengthening the practices already carried out in the home according to the specific needs of the population, based on their own cultural, economic and social capacities, as the concept of *multiple literacies* proposes.

## Methods

For this qualitative study, interviews were conducted with mothers in northwestern Mexico who had at least one child between the ages of three and six who was not yet in primary school. The purpose was to inquire about mothers’ beliefs about initial literacy and how these beliefs are expressed in the home. A total of 102 interviews were administered to a convenience sample, focusing on topics related to literacy practices in the home, language stimulation, resources and spaces available for reading and writing, and the role of caregivers.

### Participants

The participants were mothers from a city in northwestern Mexico, across several localities from varied socioeconomic sectors. The mean age of the participating mothers was 33.1 years, with 18 being the minimum age and 53 the maximum. The age of the children was a minimum of 3 years and a maximum of 6 years with an average of 4.6 years. The families interviewed had an average of 2.1 children per household. Most of the people interviewed reported being married and the most common occupation was housewife, followed by employees, teachers, merchant and students. The schooling of most of the mothers was university, followed by high school, six only went as far as middle school and four reported having postgraduate studies.

There were mothers who, despite having university studies, reported their occupation as housewife. The sectors were distributed throughout the city and the coastal zone of the municipality. The area with the greatest number of participants was the northwestern part of the city, which is distinguished by the growing development of overcrowded settlements and industrial zones, some of which are located on the outskirts of the municipality, placing them in a low socioeconomic stratum, as is the case in the southern areas of the city (see *Table 1*).

**Table 1:** Sample characteristics (N = 102)

Mother age (mean = 33.1)	N	Geographical distribution	
Age < 35 years	**57**	Northwest zone	29
Age >35 years	44	Northeast zone	12
**Children age (mean = 4.6)**		Southeast zone	13
Age < 4 years	46	Southwest zone	12
Age >4 years	56	Coast	2
**No. of children (mean = 2.1)**		Occupation	
No. of children <2	65	Housewife	39
No. of children >2	37	Academy area	11
**Income ($13, 884 MXN)**		Administrative area	11
1,700–9,500	28	Arts	2
10,000–20,000	28	Merchant	4
21,000–80,000	11	Employee	3
**Mothers’ educational level**		Student	3
Middle School	6	Freelancer	3
High School	23	Industry	3
University	39	Health	8
Postgraduate	4	Public server	1

### Procedure

The interviews were conducted using a pre-designed questionnaire based on the literature. These questions aimed to explore beliefs and behaviors in households related to literacy through various aspects, including the limiting environmental factors, the literacy interface (children’s direct or indirect exposure to literacy activities), available spaces, the frequency of literacy activities, and the notions held about reading and writing. The interviews were conducted via video calls and documented through notes and audio recordings.

Following the criteria of content analysis, the information was compiled, the interviews were transcribed, and folio numbers were assigned for easier access at a later date. Subsequently, the data were coded according to a table of key word-frequencies identified for study. The categorization of these codes was established based on the creation of interview questions, as only issues related to the process, beliefs and behaviors concerning literacy in the home were addressed (see *Table 2*). A second analysis was carried out to confirm that the categories were adequate, making only some adjustments in the conceptualization of the themes. Once the coding and classification by themes was completed, a synthesis of the interpretation of the data was conducted, describing the themes that emerged in relation to initial home literacy.

**Table 2:** Interview guide and inclusion criteria and resulting categories

Categories	Codes	Example question
***Sociodemographic***		
Income	Numerical value of monthly income.	What is your monthly income?
Mother’s school level	Mother or father’s educational level.	What is your last educational level?
Occupation	Mother or father’s line of work.	Where do you work?
Residence area	The area where they reside.	In which district do you live?
***Limiting Environment***		
Available materials	Resources and materials used for R-W activities such as books, pencils, colored pencils, paper, boards, markers, notebooks, textbooks, pen, etc.	What type of writing and reading materials are commonly found in your home?
Mothers’ reading and writing ability reported by themselves	Reading and writing habits at home. Type of material used by mother or father to R or W.	How often do you read by yourself?
Attitudes towards learning	Parents show interest in their daughter/son’s education. Academic expectations of daughter/son.	Do you help your child with academic work? How?
***Literacy interface***		
Active HLE	Direct activities for the purpose of teaching to R or W.	Do you read in front of your children?
Passive HLE	Indirect R-W activities without teaching purpose. Leisure and recreation activities.	Does your child see you writing?
Maternal interest	Opportunities that could contribute to the transfer of values and behaviors of learning to R-W Dimension centered on the mother or father.	Do you think that mothers should be involved in the reading and writing process? How?
Maternal motivation	Parents’ activities aimed at fostering children’s interest in literacy. Child-centred dimension.	Do you think it is important for children to learn to read before elementary school? Why?
***Frequency***		
Shared reading frequency		How often do you read to or with your child?
Time dedicated to school activities		How many times per week do you engage in such activities?
***Beliefs***		
Importance of reading	Parents’ views on the role of reading in fostering personal growth and development.	What do you think is the best strategy for your child to learn to read and write?
Reading as support to other areas of knowledge	Parents’ perspectives on reading as a foundation for supporting other areas of knowledge.	Do you think reading is a support for learning mathematics or science?
***Physical environment***		
Reading, writing or study areas	Areas around the house designated for R or W purposes.	What area is the most used for reading at home?
Furniture available	Furniture, lighting, and accessibility suitable for children.	Does the space have the appropriate conditions for these practices?

## Results

The results were obtained from different analyses: first, a coding of concepts was carried out, followed by a separation of themes, and finally, the different dimensions encompassing these thematic contents were named. The dimensions that emerged include, *constraining environment, literacy interface, physical spaces, frequency of activities*, and *beliefs of literacy learning*. Different themes emerged from these dimensions. The following describes the results obtained from the coding and classification of the interviews conducted with mothers according to different dimensions of the HLEs.

### Limiting environment

First, the limiting environment consists of the ability and willingness of mothers and fathers to provide reading and writing opportunities. This general theme includes subcategories such as reading and writing materials in the home, the mother’s reading skills, and both positive and negative attitudes toward literacy.

### Learning materials

All parents expressed having reading or writing materials at home for either academic or entertainment purposes. The most frequently mentioned reading materials are story books, magazines, textbooks, and newspapers:

**I006: “***Most of them* (materials) *are story books, Santiago hardly likes to read.”*

**I020**: “*We have some story books that have been of interest to him since he has chosen them when we have gone to the supermarket, they have many images and that is what draws his attention the most.”*

**I045**: *“The same books and notebooks as his older brothers, dinosaur books for the middle son, and he really likes to draw.”*

To a lesser extent, encyclopedias, novels, dictionaries, comic books and digital devices were mentioned. It should be noted that no mention was made of everyday items such as clothing labels, food packaging nor cleaning or hygiene products. The most frequently used writing materials were notebooks, sketchbooks, sheets, boards, and textbooks:

**I005**: *“He has notebooks and blackboard at home. At his grandmother’s house he has more notebooks to write.”*

**I032: “** *There are notebooks at the house, both mine and my wife’s and her own notebooks.”*

**I047**: “*He has a mini notebook where he writes his name and words that I dictate to him on occasion.”*

**I085: “** *Lined notebooks for beginners, with guides to learn how to write.”*

The least frequently mentioned were colored pencils, markers, or crayons, which were primarily considered drawing tools. Respondents did not associate these with literacy practices:

**I052**: *“Well, there in his things he has a writing board, he has paper and crayons. He doesn’t have pencils yet.”*

### Maternal skills

In exploring maternal skills, we inquired about language use and reading practices within the home. Shared reading emerged as the most frequently mentioned activity, mostly as academic support when mothers assisted with preschool assignments. Some parents were also pursuing their own studies at the time of the interview, making reading and writing part of their daily routine:

**I048**: *“She sees my partner reading every day, since she is studying at university, and she does homework quite often and my child notices it. I visit my partner every day and I take her with me because she really likes spending time with us. As for writing, whenever I am writing and she notices, she runs to sit next to me and watches carefully as I write, I think she is curious to see the pencil move faster than she can move it.”*

**I049**: *“She sees me reading every day because I am coursing a technical degree, and I have a lot of theory to read. Every time she sees me sitting in the living room she sits down and takes a notebook and imitates what I do. I don’t usually write in front of her because she always wants to take the instrument I write with and start writing herself.”*

Some parents do not perceive the use of digital devices as reading, nor are they certain that their children recognize this form of activity as a literacy activity:

**I052**: *“I often don’t read physical books because I usually read a lot on my computer or phone from time to time, and rarely a physical book, but I don’t know if he knows that we’re reading... I honestly hardly write on paper, because I don’t need to, almost everything is on the computer. I don’t see myself with a pencil and paper, it’s very rare, unless I am signing a document or something.”*

Another form of shared reading is during thebedtime routine, when mothers often read stories to their children; however, the mothers carry out the activity while the children play a passive role. There is no mention of asking questions or having the children try to guess what will happen next in the reading:

**I015: “** *At the end of the day we read a bedtime story together or at certain times during the day we read stories that interest him.”*

**I058**: “*Every day we try to read them a story, it depends on how many times they want us to repeat it (laughs) and I don’t know how often but I think their grandmother also reads to them daily when she takes care of them.”*

On the other hand, language stimulation is considered by many mothers and fathers as the correction of phonetic errors or language use. It is important to note that another common response is to play word games, such as singing, saying rhymes and riddles, and spelling words. Despite this, it is observed that activities related to academic teaching are favored in the family environment. In addition to the strategies already established above, another way mothers stimulate language is through the help of tutors, therapy, or motor exercises.

**062**: *“Look, I feel like it took him a while to talk and a friend of mine who is a psychologist has taught me things like putting honey on his lips and using his tongue to remove the honey from around them, putting a pencil in his mouth doing exercises, things like that. I’ve never really taken it because I don’t feel like it’s something so serious, it’s just that it’s his age that’s making him take a little longer to talk.”*

**I064** *: “Well, he has ADHD so he has a special teacher who gives him activities so he can speak better, so that’s what we do.”*

**I065**: “*Yes, the child has a language delay for his age, which is why he goes to therapy so that he can develop language and also at home certain exercises are carried out so that the language can be more fluid and teach him new words.*”

### Attitudes toward literacy

This category indicates favorable attitudes toward HLEs, expressed through explicit and implicit comments about mothers’ and fathers’ opinions about teaching literacy at home and the importance of education in general. More than 70 percent of respondents state that they are interested in their children learning to read and write and that mothers and fathers should support the literacy development process. However, attitudes of disinterest or misinformation about HLE are also present, ase they allude to the idea that school activities alone are sufficient for the development of reading and writing, with little need to engage in these activities at home. In other words, mothers and fathers believe they should wait until their children enter primary school to learn to read and write, as teachers are better equipped to teach literacy skills, and parents should only assist with homework.

**I013**: *“Yes, I would like him to know that* (read and write) *when he comes in* (to school) *so that he doesn’t get delayed for any reason.”*

**I049** *: “I think she is still very young, right now I am more interested in her learning to distinguish more dynamic things like colors, letters and numbers.”*

**I052**: *“Well, it would be good. I don’t think it’s necessary because that’s what school is mainly for, but he’s been practicing numbers and letters, he’s already familiar with them. What’s missing is that in his language class they teach him the syllables and all that, and I imagine that while he’s learning that, he’ll reaffirm that, since he already has the mechanical part in his little hands of knowing how to draw letters and numbers, it will become easier for him to tie all that together so he can start writing sentences and so on. I imagine that that will come along with the classes.”*

This subcategory also includes comments that refer to waiting for a certain age to begin the literacy process because they do not want to pressure their children to carry out activities that they feel they are not yet ready for.

**I002**: “*Yes, on the one hand, but I wouldn’t want to force him into something that takes its time.”*

**I033***: “If he can and have the capacity, of course yes. I would like him to do so* (read and write before elementary school)*.”*

**I048** *: “Of course I am interested, but I don’t plan to pressure the girl. I think that being consistent in her practices and doing the activities that her teachers give her is more than enough, she will do it on her own in her own time.”*

### Literacy interface

The next major theme is the literacy interface, which refers to activities carried out at home that facilitate reading and writing practice, such as creating spaces that encourage access to literacy materials and resources. The subcategories are *active HLE, passive HLE, parental interest and motivation*. Parental interest are the opportunities that potentially contribute to the transfer of values and behaviors conducive for literacy processes. On the other hand, parental motivation encompasses all activities aimed at fostering children’s interest in reading and writing; therefore, it is a child-centered dimension.

### Active HLE

Active HLE are activities designed to direct the teaching of reading and writing by parents. It has been observed that the promotion of reading is common in the homes of interviewees. As mentioned above under maternal skills, the activities most related to this dimension are shared reading, mainly at night before bedtime or during the children’s homework, as well as language correction. Similarly, in attitudes towards literacy, it was established that the focus is mainly on academic or explicit teaching. As a result, efforts are centered on the adequate use of vocabulary, proper pronunciation of phonemes, and syntactic and grammatical corrections:

**I049: “** *Sometimes we put into practice some exercises that we find on the internet to improve his pronunciation, since he is only 4 years old and some words are difficult for him, especially those that have rr.”*

**I052**: *“Yes, I try to pay close attention when he doesn’t say a word correctly, so as not to scold him, but to correct him, and tell him that it is said “in such a way” but without getting angry and without scolding him. He is already used to it and does not get sad, he knows that I am teaching him.”*

**I054**: *“Talking to him, correcting him when he makes a mistake when saying a word: “Ya sabí”, instead of saying “Ya sé” (I know); he is corrected, but in a good way so that he knows how to express himself and what words he should use when he is trying to explain something or talking about something.*

**I090**: *“I write words in notebooks and have him repeat the syllables with me and that is helping him a lot now he says the words more completely and he doesn’t stutter when he is pronouncing something.”*

### Passive HEA

In contrast, passive HEA are those common reading and writing activities that have no intentional teaching purpose performed by adults, sisters, brothers, tutors, or anyone else with whom children have contact within the home. The development of reading and writing at home mainly occurs when one of the caregivers work at the home or is currently studying. Likewise, it was found that in some homes, there is little to no modeling, even though they report that children can observe mothers read and answer messages or comment on social networks on cell phones, tablets or computers.

**I005: “** *I don’t read a lot. I only use the cellphone during the day. I usually send messages and sometimes I comment on social media. I also read the news on the phone”*

**I047**: “*Well, I’m not much of a reader, obviously I read, but only on Facebook, and I don’t read out loud.”*

They also mention that younger children seek assistance from siblings or other relatives with whom they have direct contact:

**I010**: *“He doesn’t see me, but he is always curious to see how a word is spelled, but he asks his older sister directly”.*

**I058**: *“His grandfather is the one he sees reading the most. He reads the newspaper every day.”*

### Parental interest

Parental interest refers to observing and taking advantage of opportunities that could contribute to the transfer of values and learning behaviors. This is a dimension focused on mothers’ and fathers’ beliefs about HEA and is identified through comments in which they express the importance of literacy and indicate support for it taking place at home. As previously mentioned under other subcategories, the main interest related to literacy is focused on the effectiveness of language use and academic support, considering it a tool for learning and communication.

**I002**: “*It* (reading) *would help him to be more expressive, and more social, and to develop more in the classroom.”*

**I010**: *“First of all, it will help you have a better pronunciation and identify how a word is written.”*

**I013***: “I think these two concepts* (reading and writing) *are very necessary both to be able to solve problems, and to want to write anything, but also to learn to express oneself because the range of words becomes larger.”*

This parent acknowledges modeling as crucial to the literacy development process, emphasizing the importance of providing access to both printed and digital reading materials and engaging in literacy practices that involve content analysis and interpretation:

**I034** *: “I really like reading, so my child sees me doing it. I show him magazines so that he gets a little more involved in this, but I prefer to put audiovisual content on digital platforms that have subtitles so that he can analyze and interpret them.”*

### Parental motivation

The parental motivation sub-code refers to the activities of either mothers and fathers aimed to foster children’s interest in literacy; these are manifest through expressions of support and encouragement, showing interest in the topics their children talk about, and demonstrating the patience and openness to listen to childrens’ efforts to explain a topic to them. This subcategory also refers to the childrens’ freedom of expression and the bonds of trust established between mothers and children that enable open communication.

**052**: “*Well, I find it very funny that he is curious about certain things and topics, and I always try to answer him according to his age. Sometimes I give him half-explanations because there are many things that he is very young and will not understand, but I do try to explain to him as much as possible so that he can understand them, and if he still has questions, I try to answer them willingly.”*

**I062:** “*Well, I think it has a lot to do with the example we give them and motivating them to do it, I think that is very important, and also providing them with material that a 5-year-old child would like, puzzles, stories, different educational materials that help them and get them interested in wanting to read.”*

**I091**: “*When Renata asks me a question and I don’t know the answer, I tell her I’ll answer later and I ignore her a bit, evading the question. However, afterwards I take the time to research what she asked me so I can answer her; many times, I answer the first thing that comes to mind.”*

### Physical environments

Regarding the physical environment of the home for reading and writing, it was found that most homes have areas for reading. However, these spaces are often adapted from rooms already assigned to other activities such as the living room, dining room or bedrooms. As a result, distractions are common during reading or writing activities. In this respect, answers were considered contradictory.

**I009**: “*It depends a lot on the time of the day, if you try to read very late there probably won’t be any free space.”*

**I010** *: “She has her own space, it’s a special area for her with her own desk.”*

**I013**: “*There is a special room like an office that also serves when we want complete silence to review or study things.”*

**I045: “** *Even with three children at home, there is a time when it is just a matter of homework, outside of that time there may be some noise both internally and externally, but not so much that you cannot read.”*

### Beliefs

Parental beliefs about early literacy encompass views on the importance of reading and writing before formal education and how these skills lay a foundation for learning in other academic subjects and contribute to overall personal development. Many parents recognize that fostering literacy skills early on has a lasting impact on their child’s ability to succeed across various areas of study and in everyday life. These beliefs also reflect parents’ ideas about the most effective methods for aiding their child’s literacy journey. Some parents see themselves as central to this process, actively seeking information and resources on strategies for teaching, supporting, and enhancing their child’s reading and writing abilities. By staying informed and involved, these parents strive to create a home environment that encourages literacy growth, addressing their children’s unique learning needs and supporting their academic development:

**I019:** “*I try to take some time during the day to read stories, most of the time they are digital stories since on the Internet there are pages with interactive stories that really catch the child’s attention, also when we walk down the street I try to get him to read short words that appear in advertisements, or even he himself takes the initiative.”*

**I020**: *“I don’t think school is enough, it is also important for children to reinforce what they have learned at home with their parents.”*

**I058**: *“You are never satisfied, so yes, I like to look for different ways in which I can help them and keep them entertained, but one runs out of ideas quickly. I try to support myself with ideas from relatives of mine who are teachers or things that I see on the Internet, but one always feels that one can do more things.”*

In contrast, some mothers openly recognize their limited understanding of early literacy development and often rely on the teacher’s expertise to guide their children’s learning journey. Rather than taking an active role in selecting or implementing strategies for reading and writing, they trust that the teacher’s knowledge and experience will provide the most beneficial approach for their children’s literacy growth. These mothers believe that, without specialized training, their involvement may not be as effective and, therefore, choose to follow the teacher’s recommendations closely, confident that this approach will best support their children’s educational progress:

**I086**: *“First, what the teacher says in class and then put her in an extracurricular course.”*

**I093**:*” Yes, for example, with the letters that my daughter is eager to read, but the teacher told me that it should be little by little, and I have doubts about whether it is bad for me to get ahead of myself in the process of her wanting to read and teach her little by little.”*

## Discussion

The results of this study reflect the practices and beliefs of mothers and fathers presiding in a city in northwestern Mexico. First, it is observed that resources are continuously available in the home for children to read and write, thus encouraging interaction with printed materials, exploration of language individually and with a family member, and personal expression. This has been shown to have a significant correlation with the development of early literacy skills, especially in terms of vocabulary growth (Bojczyk et al., 2016; Inoue et al., 2020; Niklas et al., 2016; Silinskas et al., 2020).

However, despite the availability of resources, these are not always appropriate or appealing to children, which can lead to poor interactions in reading and writing, as mothers and fathers tailor their beliefs about literacy based on their own experiences as noted by Bojczyk et al. (2016). Karpava (2021) points out that the choice of teaching or exposure activities can be based on parents’ school level and experiences, family socioeconomic status and cultural background which are factors that influence teaching activities and language exposure; so, the richer the HLE, the more opportunities for literacy development children will have.

It is relevant to note that few families indicated that computers, tablets, or cell phones are sources of language exposure despite having at least one of these devices in the households. In this respect, Kotrla Topic et al. (2020) state that children whose parents have lower levels of education and participate less frequently with their children in activities that stimulate literacy, may be especially susceptible to the negative effects of excessive use of digital media for entertainment purposes. So, it is recommended that these parents be encouraged to participate more frequently in interactive reading, which is found to be positively correlated to early literacy development. In agreement with this view, Attig and Weinert (2020) indicate that no single aspect of parental behavior is associated with children’s language development, but that a variety of dimensions are related to child language development, even if contributing only an incremental effect on early childhood development. The authors found that the quality of parental interaction behavior and the frequency of joint activities varied by socioeconomic status, whereby mothers with a lower socioeconomic status (SES) interacted with their children less sensitively, in less stimulating ways, and less frequently than mothers with a higher SES.

Another finding is that maternal beliefs about language stimulation refer to strategies related to explicit teaching activities or those focused on a formal and academic environment, as well as time-sharing and modeling, though to a lesser extent. Studies such as those by Guo et al. (2021) and Inoue et al. (2020) point out that parent-directed teaching of reading skills and formal activities are not significant predictors of a child’s initial writing success but are primarily associated with grammatical knowledge and the structure of printed material. On the other hand, shared reading by mothers and fathers has been shown to have a strong influence on language and literacy development, though not all reading practices yield similar results.

The study conducted by Batista Rocha and da Mota (2022) indicates that the reading style adopted by families when reading to their children produces different results in the development of pre-literacy skills. This study suggests that reading the text faithfully with pauses for explanations about the meaning of the text provides greater benefits in the development of phonological awareness and vocabulary skills. Additionally, contact with a new word, even if the child does not initially understand its meaning, expands vocabulary and, with subsequent explanation of the meaning, also improves comprehension skills. During the interviews for this research, a low percentage of families mentioned pausing when reading with their children.

In relation to the belief in exposing children to early literacy practices at home, mothers and fathers expressed an interest in engaging in activities to stimulate their daughters and sons. However, many stated that they did not know how to create a favorable HLE and instead focused, as mentioned earlier, on academic exercises aimed at correcting young children. The findings of the study by Niklas et al. (2016) indicate that starting early supports the development of children’s language skills and that the onset of shared reading is a strong indicator of general HLE. Consequently, parents should be encouraged to start reading to their children when they are very young - the earlier the better.

Finally, the findings of this study indicate that parents often view children as passive individuals, believing they lack the ability to express meaning through their doodles. As a result, parents primarily focus on conventional graphic representations of language. As promulgated by Batista Rocha and da Mota (2023); Incognito and Pinto (2023); Justice et al. (2020); Sajawandi et al. (2021) and Tsirmpa et al. (2021), more research is needed on parental beliefs as this may influence their behaviors towards their children and their ability to create linguistically enriching spaces. Additionally, Sajawandi et al. (2021) and Batista Rocha and da Mota (2023) highlight the importance of promoting reading so that children are not only taught to read but are encouraged to cultivate the taste and culture of reading.

## Conclusion

The beliefs of mothers and fathers in this region of Mexico regarding early literacy are favorable for the creation of HLE, as they express interest in having their daughters and sons engage in reading and writing from infancy. Studies indicate that mothers with interest in early literacy tend to provide greater support to their children in the literacy development process (e.g., Hume et al., 2015; Kumalasari & Sugito, 2020; Husain et al., 2011). The establishment of affective bonds between parents and children is emphasized, as it helps children feel comfortable and free. This, in turn facilitates their expression and provides opportunities to explore language without fear of reprisals. On the other hand, constant corrections of word use or correct articulation of phonemes can be detrimental if used as the sole source of stimulation and interaction with spoken and written language leading to elevated, fatigue and diminishment of their willingness to express themselves and possibly instilling problems of self-concept or impairment of language skills. This critical period of cognitive and emotional development has long-term implications, as suggested by Carneiro et al. (2019), further highlighting the need to provide an environment conducive to the comprehensive growth of children in their relationship with language. Findings such as those of Kong and Yong (2023) and Wirth et al. (2022) highlight the relevance of satisfying not only the academic components of language but the importance of establishing a safe and trusting environment where they can freely explore language use and skills. The incorporation and practice of specific behaviors that increase parent-child interaction may produce greater outcomes not only for vocabulary but also for related early literacy skills to school readiness. Therefore, parents may also improve children’s future academic success upon entering school (Dicataldo et al., 2022).

Another important aspect to highlight is the ongoing confusion or lack of information regarding what early literacy entails, the potential it holds at this stage of development, and how to foster spontaneous situations that encourage the use of language and a natural approach to reading and writing. This confusion often stems from outdate notions about literacy, such as the idea of readiness or the idea that literacy is solely achieved through maturation. Tsirmpa et al. (2021) point out that parents with conventional ideas about literacy have the belief that school is primarily responsible for teaching children. They view the development of literacy skills a result of code-based activities and often fail to recognize the significance of oral language or the role of reading in improving literacy.

As demonstrated in the studies (Batista Rocha & da Mota, 2023; Incognito & Pinto, 2023; Justice et al., 2020b; Sajawandi et al., 2021; Tsirmpa et al., 2021a), more needs to be learned about parental beliefs as it can influence their behaviors towards their children, as well as the skills necessary to create linguistically enriching spaces. Additionally, Sajawandi et al. (2021) and Batista Rocha and da Mota (2023) highlight the importance of promoting reading so that children are not only taught to read but are encouraged to cultivate the taste and culture of reading.

For the creation of a maternal intervention program focused on teaching of strategies that promote literate environments at home, it is recommended to begin by deepening of the beliefs of the target population. This approach ensures that the program addresses the particular needs of that group and establishes an action plan aligned with their lifestyles, available resources, and social configuration. Reinforcing beliefs about this process can strengthen parents’ awareness and understanding of how a rich HLE contributes to their children’s early and natural literacy development.

## Limitations

Some limitations encountered in conducting this study relate to control over the interviewers. Despite receiving prior training to conduct the interviews, variations in their approach may have affected the precision or length of the responses. However, the material obtained from the questions was deemed sufficient to achieve the study’s objective.
